# Effect of storage on the nutritional and antioxidant properties of brown Basmati rice

**DOI:** 10.1002/fsn3.2962

**Published:** 2022-07-18

**Authors:** Aqsa Naveed, Muhammad Zubair, Ayesha Baig, Mujahid Farid, Waqar Ahmed, Rafia Rehman, Muhammad Adnan Ayub, Abdo Hassoun, Janna Cropotova

**Affiliations:** ^1^ Department of Chemistry University of Gujrat Gujrat Pakistan; ^2^ Sustainable AgriFoodtech Innovation & Research (SAFIR) Arras France; ^3^ Department of Environmental Science University of Gujrat Gujrat Pakistan; ^4^ Department of Chemistry University of Okara Okara Pakistan; ^5^ Department of Chemistry University of Sahiwal Sahiwal Pakistan; ^6^ Syrian Academic Expertise (SAE) Gaziantep Turkey; ^7^ Department of Biological Sciences Alesund Faculty of Natural Sciences Norwegian University of Science and Technology (NTNU) Trondheim Norway

**Keywords:** antioxidants, nutritional, rice, storage, temperature

## Abstract

The purpose of the present study is to evaluate the effect of storage time and temperature on the nutritional and antioxidant values of different varieties of brown rice. PARB approved indigenous Basmati varieties (Basmati 86, Basmati 515, Basmati super, Basmati super fine and Basmati kainat) were procured and initially tested for physicochemical parameters, including moisture, ash, lipids, proteins, carbohydrates, and fibers from the brown rice powder. Similarly, antioxidant capacity of these brown rice samples was assessed in terms of total phenolic content and 2,2‐diphenyl‐1‐picrylhydrazyl radical‐scavenging potential. Samples of brown rice were stored for 3 and 6 months at 25 and 5°C. On increasing the storage time and temperature, antioxidant activity of rice decreases up to 50%. Nutritional parameters, such as minerals, carbohydrates, and fatty acids were characterized using UV/Vis spectrophotometer, ICP‐OES, GC–MS, and HPLC, revealing significant changes in the chemical composition of brown rice. Observation indicates that storage at high temperatures leads to a rapid decrease in carbohydrate and moisture content than at lower temperatures. The protein and ash content remains controlled and integrate with the mineral composition found. Decrease in the glucose and fructose amount was observed in brown rice varieties except for Basmati super fine and Basmati kainat at 5°C. Regarding fatty acids, oleic and linoleic acids were prominent in oils extracted from the different brown rice varieties, and their content was reduced during the storage due to conversion to behenic, and erucic acids, respectively. From the present study, it can be concluded that low storage temperatures reduce the loss of nutrients, offering better nutritional quality for the consumer.

## Graphical abstract

The influence of storage on nutritional properties of Basmati rice.

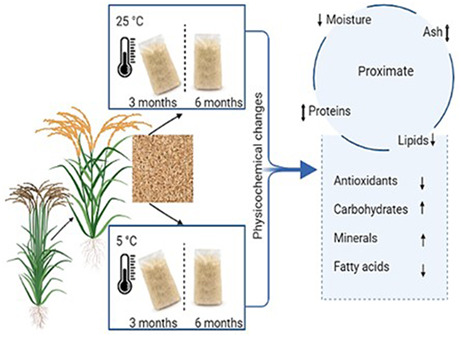

## 1 INTRODUCTION

Paddy rice consists of husk and brown rice, which is refined to polished white rice and bran. Brown rice has the structural composition of pericarp (1–2%), aleurone, subaleuron and nucellus (4–6%), embryo (2–3%), and starchy endosperm (89–94%). The aleuronic layer consists of 1–5% cell layer (Nawaz et al., 2018), while the rice bran layer and embryo contain large amounts of ash, and gamma oryzanol, tocopherols, tocotrienols, sterols in lipids contents comprising saturated and unsaturated fatty acids (Moongngarm et al., 2012). Regarding the fatty acids profile, rice bran usually comprises unsaturated fatty acids, such as linolenic, oleic and linoleic acids, and saturated ones including predominantly palmitic and stearic acids (Muhammad, 2011). Minerals, especially phosphorous (P), manganese (Mn), potassium (K), magnesium (Mg), calcium (Ca), and sodium (Na) are also present in rice grains (Kalpanadevi et al., 2019). In addition, many heavy metals, such as iron (Fe), aluminum (Al), sodium (Na), silicon (Si), and zinc (Zn) are found in the rice bran (Lin et al., 2020; Zhou et al., 2015). Phytochemicals, for example, homologues of vitamin‐E (tocopherols and tocotrienols) and gamma oryzanol are major antioxidants in rice (Bhattacharya, 2017). The rice bran is also a rich source of phenolic acids; rice has the highest concentration of free phenolic acids after corn, whereas the antioxidant capacity of rice is less than the other grains due to its lower concentration of bound phenolic acids. Therefore, consumption of brown rice is more beneficial than that of milled rice due to its nutritional superiority (Van Hung, 2016). Consumers of different countries have various preferences toward freshly harvested and stored rice. Freshly harvested rice is preferred in China, Korea, Vietnam, and Japan due to their stickiness while stored rice is liked in subcontinent and Middle East countries (Pratiwi & Purwestri, 2017). Rice grains can be stored in paddy or milled forms. Storage of paddy (rough rice with husk) reduces loss of nutrients and grain quality while storage of milled rice is more convenient economically and requires less space, making it more attractive for grocery stores and supermarkets in cities (Ahmad et al., 2017). Appropriate storage conditions are important to avoid spoilage, degradation, and germination of dry rice. However, the long journey of rice “from farm to table” involves changes in the physical and chemical properties of brown rice, leading to modifications in the nutritional value. Thus, the commercial value of the rice grains is influenced by aging process and storage conditions (Devraj et al., 2020). Protein, starch, and lipid contents could drop at considerable rates at higher temperatures storage. Increase in bitterness and considerable worsening in the taste and flavor of rice occur due to production of free fatty acids or peroxides from hydrolysis and oxidation of fatty acids and carbohydrates. As the albumin decreases, the solubility of protein could be reduced even if protein contents remain unchanged. Storage of rice results into the loss of free amino acids of outer layer of rice grain which causes the browning of the rice. Furthermore, enzymatic activities could occur in stored milled rice including decreases in amylase and increases in protease and lipase (Devraj et al., 2020). Storage temperature and duration play significant role in variations of nutrients of rice. Storage temperature could affect enzymatic activities, influencing protein, starch, and lipid contents of stored rice (Ojha et al., 2018). A conceptual model gave information about interaction between starch and non‐starch components (lipids and proteins) in the endosperm cells of the old rice (Zhou et al., 2015). This mechanism suggested that protein molecules undergo chemical reactions altering structure and thus properties of proteins (Kraithong et al., 2018). These days' researchers prefer to maintain nutrients quality in whole grains during storage for the satisfaction of the consumers. Although some research studies on the characterization of chemical nutrients of Basmati rice cultivars have been conducted, there is still a gap of information on the compositional changes that occur during storage. Therefore, the present project was designed with the prime objective of comparative profiling of nutritional variations occurs during the storage of Basmati (aromatic) brown rice of different varieties.

## 2 MATERIAL AND METHODS

### 2.1 Collection, pretreatment and storage of brown rice samples

Paddy samples 5.0 Kg each of approved varieties from Pakistan Agriculture Research Board (PARB), Basmati 86, Basmati 515, Basmati super, Basmati super fine and Basmati kainat were procured from Directorate of Rice Research, Kala Shah Kaku District Gujranwala, Pakistan. Samples of paddy rice were dried in the sun to make it suitable for storing to prevent microbial contamination. Each sample (of 5.0 kg) rice of each variety was equally divided into five parts. One part was used for the initial physiochemical study and the other four were packed in polythene bags and were stored in two groups of storage (3 month and 6 month) as well as temperature (5 and 25°C) as shown in Table 1.

**TABLE 1:** Research design

Stages	Seasons & time	Temperature	
Initial stage	After sample procurement	–	–
1st storage	November–February (3 months)	5°C	25°C
2nd storage	November–May (6 months)	5°C	25°C

### 2.2 Husk removal and grinding of brown rice

Husk was removed from all types of dry paddy rice samples at each stage (initial, 3 months and 6 months) and each temperature using Stake Rice Huller (model THO35A) to obtain brown rice. For analysis purposes, these brown rice samples were processed into a fine powder (80 mesh size) in a coffee grinder. Ground rice samples were stored in polythene bags in a refrigerator to avoid lipase activity and other physicochemical changes.

### 2.3 Determination of Physicochemical parameters of brown rice

In the present work, proximate composition is determined by a series of methods given by AOAC‐1990 and expressed as percentage (g/100 g) of brown rice. Determination of the physicochemical composition values of dehulled and pulverized brown rice samples, including moisture, ash, crude (protein, fiber, and lipid) and total carbohydrates of each phase (initial, after 3 months, and after 6 months) and each temperature was performed by using the Standard methods of analysis, such as ultraviolet–visible (UV/Vis) spectrophotometer, inductively coupled plasma optical emission spectrophotometer (ICP‐OES), gas chromatography mass spectrometry (GC–MS), and high performance liquid chromatography (HPLC) (Katsa et al., 2021; Tai et al., 2021).

### 2.4 Determination of minerals in brown rice

Minerals in brown rice samples were determined by ICP‐OES. For this, two (2.0) grams of each brown rice sample was digested in 10 ml Conc.HNO_3_ placing on a heating plate at about 80°C for 2–3 h. After digestion of solid material, it is cooled to normal state to 25°C temperature then added 5.0 ml H_2_O_2_ and heated again at 160–170°C for complete oxidation of organic matter till transparent solution obtained which was diluted to 50 ml in volumetric flask. The analysis was performed by using ICP‐OES (ARL‐3580). Accurate standard solutions of 23 elements of 1000 ppm were prepared. Calibration curves were constructed by using these prepared standard's solutions of the minerals and used for the calculation of the actual results (Muhammad et al., 2012; Shao et al., 2016).

### 2.5 Determination of simple sugars in the brown rice

Analysis of brown rice samples simple sugars (glucose, fructose, and sucrose) was performed by reverse phase high performance liquid chromatography (RP‐HPLC) model 2010 Prominence Shimadzu, Japan. Sample solution was prepared by extracting 2.0 grams of rice flour with 20 ml of ethanol: water (2:3) mixture on orbital shaker for 2 h. The extract so obtained was centrifuged and filtered. Excessive solvent was evaporated by keeping extract in an oven at 60°C. The residues were then dissolved in 0.4 M H_2_SO_4_ and filtered through 0.20‐micron filter paper and poured into sampling vials for HPLC analysis. Separation of carbohydrates was performed on a C_18_reverse phase column (300 × 7.8 mm). The elution of carbohydrates was made by using a 17 mM sulfuric acid solution as mobile phase at a constant flow rate of 0.8 ml/min. A refractive index (RI) detector operated at 40°C was used for detection purpose. Standards of pure soluble sugars were used for the identification and quantification of compounds (Zubair et al., 2012).

### 2.6 Determination of fatty acids of the brown rice

Fatty acids of all indigenous Basmati varieties (Basmati 86, Basmati 515, Basmati super, Basmati super fine, and Basmati kainat) were converted to methyl ester derivatives. An amount of 50 mg of extracted rice oil in n‐hexane was treated with 5.0 ml of methanolic KOH (0.5 M). Solution was heated at 80 °C. Reaction converted the fatty acids into FAMEs and extracted into low‐density layer of pure n‐Hexane. The layer containing FAMEs was transferred to a sample GC vial using a pasture pipette. Prepared FAMEs injected into (Splitless injection) GC–MS (Shimadzu QP 2010, SHIMADZU Corporation, Japan) for fatty acid composition. A polar capillary column (Rtx‐5MS, film thickness 0.25 μm and length 30 m × 0.32 mm ID) was used with oven temperature programmed from 170°C for 1.0 min to 290°C with a ramp rate of 5°C/min and the last hold‐up was 10 min. Helium with 99.9% purity, was used as a carrier, with a flow rate of 1.0 ml/min throughout the analysis. The mass detector was operated in electron impact (EI) mode with an ionization energy of 70 eV and a m/z scan range of 35–500. Compound detection was made using GC–MS's built‐in NIST 2017 updated library. All the samples were analyzed in triplicate.

### 2.7 Determination of Total phenolic contents of brown rice

Antioxidants were extracted from rice flour with pure methanol and acetone as well as 80: 20 methanol: water and 80:20 acetone: water on orbital shaker (Hu et al.,^1996^; Duvernay et al.,^2005^). For this purpose, 10 g of rice flour was extracted by applying 100 ml (1:10) 100%, 80% methanol, and 100%, 80% acetone at 40°C and 120 rpm for 1 day. After filtration, the extract was concentrated on a rotary vacuum evaporator. These extracts were kept in the freezer for further analysis. Samples of the control and stored selected varieties of brown rice were analyzed for TPCs using the modified method of Mir et al. (2020). Briefly, 0.1 ml of sample solution was poured into a test tube having 2.0 ml sodium carbonate (7.5%) then mixed for 3 min. 1.0 ml of Folin‐Ciocalteau reagent (10%) was added followed by the addition of 6.9 ml distilled water to make final volume 10 ml. This mixture was kept for half an hour to complete the reaction. Then absorbance at 760 nm was recorded by UV–Vis spectrophotometer. Gallic acid was used as reference standard and concentrations are reported as GAE/kg.

### 2.8 DPPH radical inhibition potential of the brown rice

Following the procedure used by Zubair et al. (2012) extracts from brown rice samples were tested for DPPH radical‐scanning activity. A 0.1 ml of sample solution was poured in a test tube having 4 ml of pure methanol. Then 1.0 ml DPPH solution (0.001 M in methanol) was added. This mixture was allowed to stand for half an hour then the absorbance was noted at 515 nm using UV‐spectrophotometer (Park et al., 2020). Calibration curve of butylhydroxytoluene (BHT) was developed for the calculation of results of scavenging activity. Absorbance of each mixture was noted thrice.

### 2.9 Statistics

All the samples analyzed in triplicate and mean were calculated. Repeated measures ANOVA was used to identify the significant variations (5%) in sugar contents of rice varieties with different storage conditions. The Tukey HSD test was then applied on those groups where a significant difference was observed.

## 3 RESULTS AND DISCUSSION

### 3.1 Effect of storage on the Physicochemical composition of the brown rice

**FIGURE 1:**
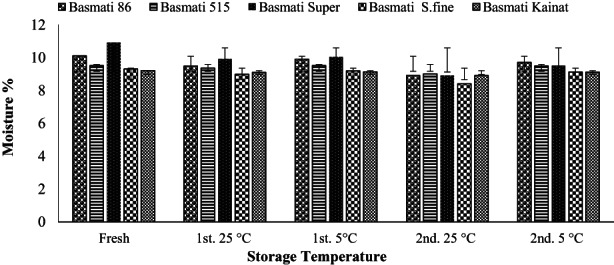
Effect of storage on moisture (average %) of brown rice of different varieties

Physiochemical analysis of brown rice of different varieties describes the content of wet (moisture) and dry matter having organic (protein, fibers, carbohydrates, and fats) and inorganic composition (ash). Fresh moisture contents measurement reveals Basmati kainat has the lowest (9.20%) moisture than all varieties which is found in close agreement with the research work in Pakistani on the rice varieties resulting Basmati kainat has the lowest moisture among all the selected varieties (Jamal et al., 2016) while the highest moisture contents observed in Basmati super (10.90%) as depicted in Figure 1. Generally, less than 12% moisture content is recommended for storage of rice to avoid attack of microorganisms and insects while about 9.19–11.1% moisture is reported in the literature for different Pakistani rice varieties (Chavan et al., 2018). The results of our study indicated that storage at the higher temperature at 25°C reduced moisture contents more rapidly as compared with the lower temperature 5°C which is earlier observed and in close agreement with other findings (Ahmad et al., 2017b). Loss of moisture contents during the storage of 3 months was found to be less than that of the second storage for 6 months which is probably due to comparatively cold weather during first storage than that of second stage. Variations in moisture content may be the cause of changes in physicochemical parameters responsible for nutritional quality of rice. Standard deviation among the samples of fresh and stored brown rice depicts more change at longer storage at higher temperature.

**FIGURE 2:**
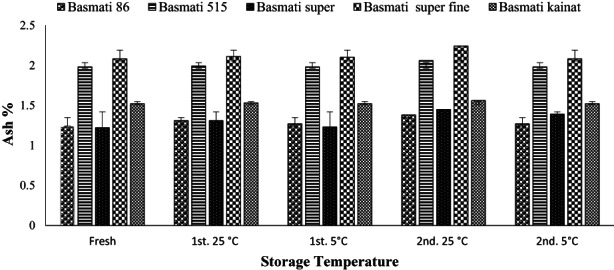
Effect of storage on the ash contents (average %) of brown rice of different varieties

Fresh brown rice samples were evaluated for ash contents which found to be varied from 1.22–2.08%, is higher than the reported earlier in the range of 0.52–1.15% in white Basmati rice (Kraithong et al., 2018). Basmati 515 and Basmati super fine comprising more minerals as depicted in ash contents whereas Basmati 86 and Basmati super have lowest contents. Observations of the storage effect indicate that ash remaining almost same, although a slight increase is noticed in samples stored at 25°C temperature as compared with those stored at 5°C, as shown in Figure 2. No any significant variation seems present in the ash contents of the brown rice as shown by the standard deviation application among the samples of fresh and stored brown rice varieties. Both temperature and time have not effect on the ash except slight increase which is due to the loss of moisture while storage. These variations are in line with previous findings (Islam et al., 2021) where similar variations of ash content after storage in refrigerator and at 25°C temperature were noted.

**FIGURE 3:**
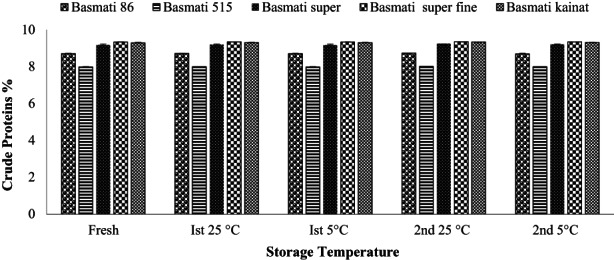
Effect of storage on crude proteins (average %) of brown rice of different varieties

Evaluation of crude protein contents of different brown rice varieties in fresh and stored samples is 7.98–9.33% as shown in Figure 3, which is slightly higher than reported values (7.1–8.9%) in white Basmati rice (Kraithong et al., 2018). Present research findings are in accordance with the previous research (Park et al., 2020) where 7.95–9.52% protein contents were reported in brown rice which is slightly higher than our results of brown Basmati rice. The results show that Basmati 515 variety has the lowest protein content, while Basmati super fine has the highest. Comparative values of crude protein are shown in Figure 3, indicate no significant variation after storage, in accordance with earlier findings (Shi et al., 2017; Yu et al., 2017). Both time and temperature as chosen for the storage do not effect significantly on the protein contents which is good sign for restoring the nutritional values of the stored brown rice. Statistically standard deviation among the varieties analyzed freshly after storage for crude proteins showing no significant variation. However, at lower temperature and short time of storage even safer than high temperature and longer time.

**FIGURE 4:**
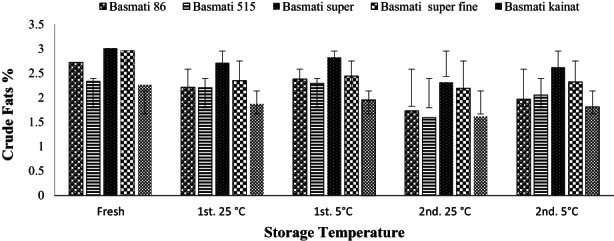
Effect of storage on crude lipids (average %) of brown rice of different varieties

Figure 4 showing the results of crude lipid contents of fresh and stored samples of the brown rice varieties ranging 2.26–3.01% with highest lipid/oil in Basmati super and lowest in Basmati kainat. High temperature cause lipid destruction in membranes and result into liberation of free fatty acids. Cold storage of rice does not affect the lipids extensively whereas hydrolysis and oxidation increases with increasing storage temperature (Sivakamasundari et al., 2020). The lipid contents found in the selected brown rice varieties are higher than those observed in the earlier research in the range of 2.06–2.60% (Rashid et al., 2020). Present study shows that storage temperature and duration has significant influence on the percentage of lipid contents, since the lipid contents decrease linearly with increasing storage time and temperature. Changes in the lipid contents are well understood after looking at the application of standard deviation in Figure 4 variations are more pronounced after 2nd storage at 25°C. Maximum loss of lipids observed in Basmati 86 and Basmati kainat after 1st stage and in Basmati 515 after 2nd stage of storage at 25°C. A small decrease was noticed in all the brown rice varieties at low temperature of storage. Figure 4 shows that the loss of lipids is linked to the increase in storage time as well as storage temperature. In a similar research work, higher lipid contents were reported in rice stored at 4°C than at 37°C (Kumar et al., 2018). More than 50% loss of lipid contents occurred during storage of rice for 1 month at 60°C while 1/3 lipid contents were lost during storage at 30°C (Ahmad et al., 2017). In another similar work, it was concluded that brown rice flour produced at low‐temperature has excellent physicochemical properties (Luo et al., 2021; Zakarya et al., 2018).

**FIGURE 5:**
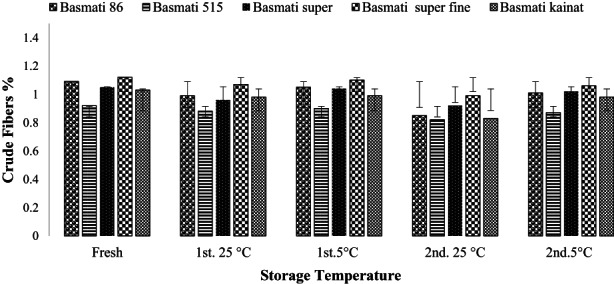
Effect of storage on crude fibers (average %) of brown rice of different varieties

Fiber contents of fresh and stored brown rice of different varieties as observed ranges 0.92–1.12% and observed highest in Basmati super fine and lowest in Basmati 515 as shown in Figure 5. Statistical analysis by standard deviation among varieties depicts that storage for long time (2nd storage for 6 months) and at 25°C results in reduction of fiber contents. Our results were in agreement with previous research where a significant effect of storage period on loss of fibers is reported (El‐Kady et al., 2013; Liu, Li, et al., 2017; Tong et al., 2019). Brown rice, a valuable nutrition, has its limitations in the industrial use due to instability during storage. There is a growing trend in the use of brown rice in the functional foods, and the consistency of nutritional values of rice bran is a prerequisite for its efficient use (Liu et al., 2019).

### 3.2 Effect of storage on simple sugars of brown rice

Three different sugars, namely, sucrose, fructose, and glucose were analyzed in the selected aromatic brown rice varieties of fresh and stored at different conditions **(**Table 2). Concentration of sucrose in the fresh samples of brown rice Basmati 86 and Basmati kainat found lower than the concentration found after storage. Comparatively, Basmati super fine has greater amount of sucrose than the other varieties. Storage period and temperature had a notable impact on the sucrose concentrations. The major increase in the concentration of sucrose of the selected varieties was noted in the samples stored at 25°C especially in Basmati super fine followed by Basmati kainat. Basmati super fine showed highest reduction at 5°C followed by Basmati 515 while sucrose increased in Basmati kainat. No variation between the other two varieties (i.e., Basmati 86 and Basmati super) was noticed for the second storage at 5°C.

**TABLE 2:** Effect of storage on the brown rice sugars contents (mg/kg)

Varieties	0 stage	1st stage		2nd stage	
	Fresh	25°C	5°C	25°C	5°C
Sucrose concentration (mg/kg)					
Basmati 86	1.50 ± 0.03^a^	3.10 ± 0.09^a^	2.20 ± 0.08^a^	17.50 ± 0.32^a^	1.70 ± 0.02^a^
Basmati 515	5.00 ± 0.16^a^	5.80 ± 0.14^ab^	2.40 ± 0.11^a^	18.40 ± 0.41^ab^	1.00 ± 0.03^a^
Basmati Super	3.10 ± 0.09^a^	3.70 ± 0.16^ab^	1.90 ± 0.07^a^	14.90 ± 0.34^ab^	3.00 ± 0.12^a^
Basmati S. fine	5.40 ± 0.13^a^	14.50 ± 0.37^ab^	1.10 ± 0.03^a^	31.00 ± 1.09^ab^	0.20 ± 0.01^a^
Basmati Kainat	1.60 ± 0.05^a^	2.10 ± 0.06^b^	1.80 ± 0.05^a^	21.50 ± 0.47^b^	4.40 ± 0.22^a^
Fructose concentration (mg/kg)					
Basmati 86	10.60 ± 0.20^a^	10.40 ± 0.31^a^	10.80 ± 0.26^a^	9.70 ± 0.34^a^	15.70 ± 0.26^a^
Basmati 515	6.80 ± 0.17^a^	1.10 ± 0.03^ab^	3.20 ± 0.12^a^	4.70 ± 0.18^ab^	3.20 ± 0.12^a^
Basmati Super	10.70 ± 0.23^a^	11.80 ± 0.27^ab^	2.60 ± 0.08^a^	9.00 ± 0.24^ab^	2.60 ± 0.08^a^
Basmati S. fine	13.70 ± 0.31^a^	9.90 ± 0.25^ab^	5.30 ± 0.17^a^	10.20 ± 0.25^ab^	7.05 ± 0.22^a^
Basmati Kainat	7.60 ± 0.16^a^	15.00 ± 0.41^b^	8.70 ± 0.33^a^	34.50 ± 1.19^b^	8.70 ± 0.21^a^
Glucose concentration (mg/kg)					
Basmati 86	37.50 ± 1.21^a^	10.20 ± 1.32^a^	4.10 ± 0.12^a^	1.70 ± 0.04^a^	1.70 ± 0.05^a^
Basmati 515	95.60 ± 2.36^a^	32.60 ± 1.50^ab^	42.40 ± 2.03^a^	28.70 ± 0.83^ab^	28.70 ± 1.10^a^
Basmati Super	54.70 ± 1.71^a^	23.20 ± 0.98^ab^	2.90 ± 0.10^a^	18.50 ± 0.75^ab^	15.70 ± 0.50^a^
Basmati S. fine	89.90 ± 3.01^a^	64.90 ± 2.22^ab^	84.30 ± 3.18^a^	30.70 ± 1.12^ab^	97.00 ± 2.30^a^
Basmati Kainat	37.50 ± 1.02^a^	25.20 ± 1.51^b^	4.10 ± 0.16^a^	27.70 ± 1.10^b^	34.50 ± 1.40^a^

*Note*: Values are mean ± SD for three samples of each variety, analyzed individually in triplicate (*n* = 3 × 3).

Means with different superscript letters with in the same row indicate significant differences (*p* < .05).

Effect of the storage at different temperatures and periods on the variation of fructose concentration is shown in Table 2. The highest fructose concentration was found in Basmati super fine while the lowest one was observed in Basmati 515. Comparison after storage stages of Basmati 515 and Basmati super fine indicates that a decreasing trend of fructose concentration occurred more at the second stage of storage at both temperatures. Basmati kainat and Basmati 86 showed exceptional behavior displaying an increase during storage at both temperatures and only at 5°C, respectively.

Effect of storage conditions on the glucose concentration is depicted in Table 2. Basmati 515 showed the highest glucose concentration followed by Basmati super fine and Basmati super, while Basmati 86 and Basmati kainat had the same concentration. It was found that in Basmati 86, a gradual decrease in glucose concentration occurs during the first storage and a significant reduction was observed after the second stage of storage. A decrease in sucrose concentration was noticed in Basmati kainat, while Basmati super fine showed a significant increase in sucrose concentration at 5°C storage after the second stage of storage. General linear model ANOVA also revealed that there was a significant difference (*p* value < .05) in sugar contents between the different rice varieties (fresh and stored at 25°C; Table S1).

These results are in close agreement with other research studies (Chmiel et al., 2018; Lim et al., 2017). A similar research study found that total reducing sugar contents increased sharply in the 16‐month storage period and thereafter, increased steadily. The rate of reduction in sugar content out of total sugar content increased with extended storage duration (Wiruch et al., 2019). In another study, Devraj et al. reported significant reductions of 26.4–64.7% and 37.1–52.1% in the content of disaccharides, that is, sucrose and polysaccharides, that is, raffinose, respectively. Significant increases of 54.0–107.1% in the content of glucose and fructose and 33.1–79.9% in the content of monosaccharides, were also reported in brown rice stored for 6 months at 25°C (Devraj et al., 2020).

### 3.3 Effect of storage on the fatty acids composition of the brown rice varieties

Table 5 represents the fatty acids composition of some selected brown rice varieties showing a significant variation in fatty acids after storage. Oleic acid has the highest composition than other fatty acids in both Basmati 86 and Basmati super varieties. A similar previous research, conducted on brown rice, showed that the oleic acid is the dominant fatty acid followed by linoleic acid and palmitic acid (Munarko et al., 2021). Effects of first stage of storage on fatty acids depicts decline in the concentration of oleic acid and an increase in concentrations of palmitic acid and appearance of new acids, that is, erucic acid, behenic acid, and arachidic acid, etc. Linoleic acid disappeared in Basmati 86 after storage of 3 months at 25°C, and appeared again during the second stage of storage, while in Basmati super, it reduces in the second stage in the samples stored at 25°C. The reduction in oleic and linoleic acids is in accordance with the previous research (Isnaini et al., 2019; Latifi & Esmaiili, 2017; Liu et al., 2021). Observing the effect of temperature on the fatty acid composition of brown rice indicates that changes in fatty acid are more significant during the storage at 25°C. Some of the newly produced acids are not found in samples stored at 5°C. This observation is in agreement with results reported in the other research studies where the reduction of fatty acids was observed due to increase in free fatty acids resulting the hydrolysis of lipids. Free fatty acids formed may be further oxidized into hydroperoxides and related products (Ahmed et al., 2016).

### 3.4 Effect of storage on the minerals concentrations of the brown rice

**FIGURE 6:**
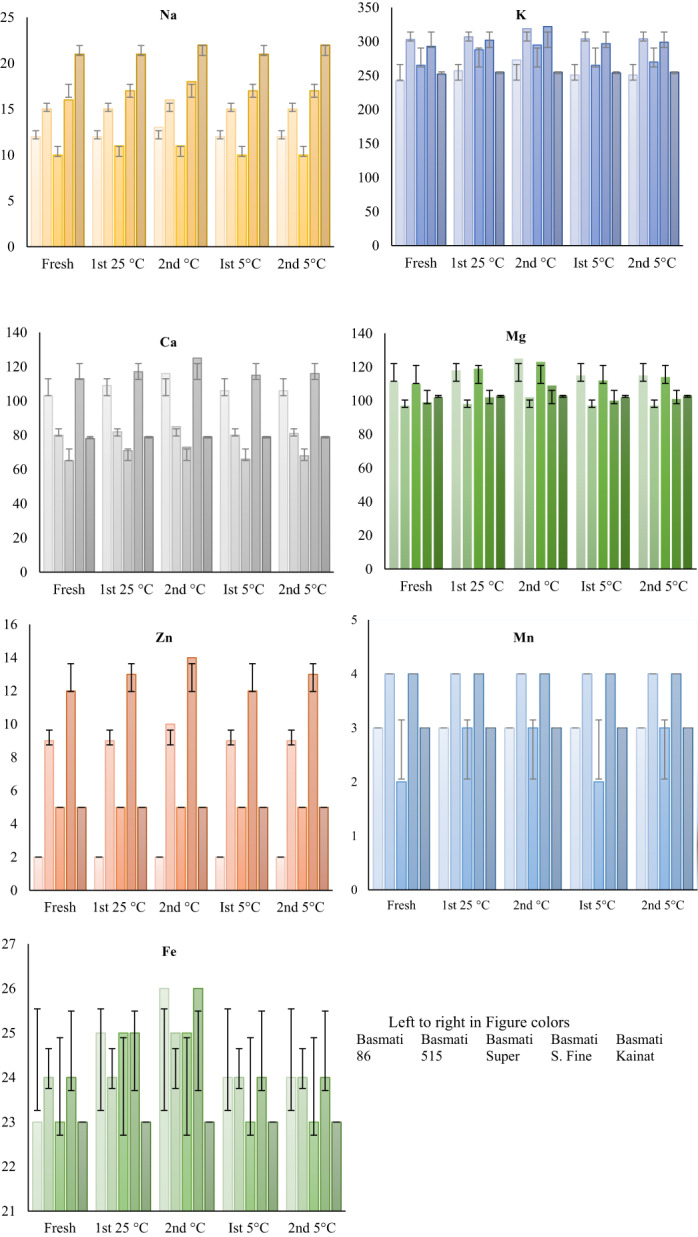
Effect of Storage on the minerals concentration (mg/100 g) of brown rice

Figure 6 shows the results of minerals of fresh and stored brown rice samples of different varieties and depicting the effect of storage on the concentration of different minerals present in the varieties of brown rice. Potassium (K) is one of the most abundant minerals found in rice followed by magnesium (mg) and calcium (Ca). Concentrations of minerals in the fresh samples of the brown rice of different varieties reveals that the potassium content is in the range 242–303 mg/100 g being the highest in Basmati 515 and the lowest in Basmati 86. An amount of 181–368 mg/100 g K is reported in a similar previous research work (Mir et al., 2020). Magnesium (Mg) is the second most abundant mineral in rice varieties, found ranging from 96.68–110.97 mg/100 g being the highest in Basmati 86. The results of present research are in close relation to other research works (Borad et al., 2017; Liu, Zheng, & Chen, 2017; Peng et al., 2019). Calcium (Ca) at an amount 113.55 mg/100 g in Basmati super fine, 65.50 mg/100 g in Basmati super and about 21.35 mg/100 g was found in Basmati kainat while 9.92 mg/100 g in Basmati super. The amount of calcium (Ca) reported in literature is found in the range of 10–50 mg/100 g which is however lower than the present work, while the amount of sodium (Na) of our study is in line with the previous finding (Kraithong et al., 2018; Somaratne et al., 2017). Other minerals including Fe, Zn, and Mn are comparable in all varieties with a higher content being observed in Basmati super and 515. In a similar research study, Liu et al. observed considerable losses of Mn, Fe, Pb, and Mg during storage and loss of elements observed is associated with the rice genotype (Liu, Zheng, & Chen, 2017). Concentrations of the other minerals were higher than those cited in earlier research probably due to increase in industrialization and metals pollution in water and soils (Ahmad et al., 2021). Our results are in close agreement with those previously reported by other researchers (Ahmed et al., 2017; Al‐Zoreky & Saleh, 2019).

### 3.5 Effect of storage on TPCs and antioxidant potential of the brown rice

Antioxidants have protective functions in the body, by controlling the free radicals through reducing or scavenging them. Free radicals are known to attack proteins, lipids, and DNA. In addition, these can act as initiating factors for many chronic diseases like cancer. The presence of phenolic compounds in the body shows various physiological effects on the human body. They prevent damage caused by low‐density lipoproteins and lipids and thus reduce the risk of coronary heart disease and cancer (Zahra & Jabeen, 2020). Phenolic compounds of cereals are interesting chemical characteristics because of their antioxidant potential and radical‐scavenging ability (Kumar et al., 2018). Hence, TPCs and DPPH radical‐scavenging ability of the brown rice were evaluated to investigate the effect of storage on the brown rice antioxidants.

#### 3.5.1 Effect of storage on the TPCs of the brown rice varieties

Totals phenolics of the fresh and stored samples of the brown rice varieties were estimated to evaluate the effect of incubation. In this research work, Basmati super fine showed the highest concentration of phenolics (1550 mg GAE/kg g) while Basmati 86 showed the lowest concentration (617 mg GAE/kg) as shown in Table 3. Elongation in storage duration results into decrease of TPC's in varieties of brown rice. A significant decline up to 70% in TPCs after second stage of storage at 25°C among the brown rice varieties in fresh and stored samples was observed. Total phenolics ranging from 0.81 to 1.64 mg GAE/g (810–1640 mg GAE/kg) were reported earlier for brown rice (Mir et al., 2016). Maximum decrease occurred during the first stage of storage (i.e., the first 3 months), especially in samples stored at 25°C as shown in Table 3. Decrease in the concentration of total phenolics after storage of brown rice was also investigated in earlier research suggesting involvement of phenolics in aging. Aging process is faster at higher temperatures leading to a more phenolics loss. Lowing in phenolic contents after storage is in accordance with a previous work (Mir et al., 2020) in which a decrease in phenolic contents was noted during 7 months from 17.17 to 6.07 and 7.29 mg/g at 25 and 37°C, respectively. There lies a significant difference (*p* value < .05) in TPCs and antioxidant attributes of different brown rice varieties after storage conditions (fresh, stored at 25 and 5°C). A similar study reports the accumulation of phenolics during thermal elicitation of rice grains and the results obtained were in close agreement with our results (Ampofo et al., 2020).

**TABLE 3:** Effect of storage on the brown rice total phenolic contents (mg GAE/kg of rice)

Varieties	Fresh	1st stage		2nd stage	
		25°C	5°C	25°C	5°C
TPCs (mg GAE/kg) in 100% methanol					
Basmati 86	617 ± 19^a^	382 ± 14^a^	530 ± 12^a^	228 ± 7.1^a^	364 ± 12^a^
Basmati 515	922 ± 35^b^	475 ± 21^b^	656 ± 26^a^	396 ± 17^ab^	408 ± 16^a^
Basmati super	1060 ± 32^b^	637 ± 20^b^	804 ± 21^a^	442 ± 15^ab^	798 ± 31^a^
Basmati S. fine	1550 ± 48^b^	829 ± 36^b^	899 ± 17^a^	621 ± 26^ab^	850 ± 30^a^
Basmati Kainat	817 ± 31^b^	541 ± 18^b^	439 ± 27^a^	318 ± 09^b^	400 ± 10^a^
TPCs (mg GAE/kg) in 80% methanol					
Basmati 86	570 ± 22^a^	307 ± 6.4^a^	427 ± 17^a^	278 ± 10^a^	507 ± 16^a^
Basmati 515	624 ± 25^b^	297 ± 11^b^	491 ± 15^a^	236 ± 05^ab^	417 ± 15^a^
Basmati Super	619 ± 19^b^	337 ± 13^b^	537 ± 21^a^	257 ± 07^ab^	497 ± 12^a^
Basmati S. fine	1009 ± 45^b^	507 ± 16^b^	657 ± 25^a^	386 ± 15^ab^	589 ± 21^a^
Basmati Kainat	598 ± 28^b^	327 ± 08^b^	498 ± 18^a^	288 ± 07^b^	428 ± 09^a^
TPCs (mg GAE/kg) in 100% acetone					
Basmati 86	527 ± 16^a^	287 ± 12^a^	488 ± 14^a^	226 ± 07^a^	409 ± 14^a^
Basmati 515	708 ± 23^b^	316 ± 10^b^	678 ± 20^a^	324 ± 13^ab^	647 ± 21^a^
Basmati Super	669 ± 22^b^	332 ± 16^b^	634 ± 24^a^	231 ± 06^ab^	607 ± 16^a^
Basmati S. fine	956 ± 31^b^	517 ± 15^b^	596 ± 17^a^	449 ± 20^ab^	507 ± 17^a^
Basmati Kainat	557 ± 26^b^	288 ± 06^b^	498 ± 15^a^	280 ± 07^b^	457 ± 20^a^
TPCs (mg GAE/kg) in 80% acetone					
Basmati 86	479 ± 16^b^	274 ± 10^a^	418 ± 12^a^	146 ± 03^a^	318 ± 10^a^
Basmati 515	555 ± 19^b^	298 ± 10^b^	439 ± 17^a^	204 ± 04^ab^	387 ± 15^a^
Basmati Super	741 ± 23^b^	328 ± 13^b^	290 ± 08^a^	238 ± 07^ab^	308 ± 09^a^
Basmati S. fine	817 ± 34^b^	426 ± 16^b^	537 ± 19^a^	201 ± 07^ab^	355 ± 05^a^
Basmati Kainat	498 ± 21^b^	239 ± 11^b^	406 ± 10^a^	194 ± 04^b^	297 ± 12^a^

*Note*: Values are mean ± SD for three samples of each variety, analyzed individually in triplicate (*n* = 3 × 3).

Means with different superscript letters with in the same row indicate significant differences (*p* < .05).

#### 3.5.2 Effect of storage on DPPH radical‐scavenging potential

Bioavailability and activity of phenolics is essential for their health claims; therefore, this research study further investigated and demonstrated the antioxidant properties of phenolic extracts obtained from stored brown rice. The results obtained in our study were in close agreement with those reported by Ampofo and co‐authors (Ampofo et al., 2020). Brown rice antioxidants are beneficial for health because they have the ability to scavenge the free radicals and reactive oxygen species. Percentage inhibition of selected rice varieties ranged from 41 to 63% in methanol, 27–47% in methanol: water, 41–47% in acetone and 24–35.75% in acetone: water, which are in close agreement with previously cited research (Somaratne et al., 2017). Highest DPPH radical‐scavenging ability was exhibited by Basmati super and lowest for Basmati kainat (Table 4) with the mean values ranging from 33.85 to –45.93%. This capacity decreased with the increase in storage time and temperature. After the first stage of storage this capacity was 27.38–32.18% in storage at 25°C and 32.07–39.88% at 5°C, while after the second stage, the scavenging capacity was 22.53–27.62% at 25°C and 30.90–35.79% at 5°C. This reduction in DPPH scavenging ability is in line with previous research reports where a decline of 45% was reported after storage of brown rice for 7 months (Devraj et al., 2020; Zahra & Jabeen, 2020).

**TABLE 4:** Effect of storage on the brown rice antioxidant activity DPPH radical (% inhibition)

Varieties	Fresh	1st stage		2nd stage	
		25°C	5°C	25°C	5°C
DPPH inhibition of rice extracts in 100% methanol					
Basmati 86	42.0 ± 1.2^a^	34.0 ± 0.8^a^	38.0 ± 1.6^a^	26.0 ± 0.2^a^	38.0 ± 1.9^a^
Basmati 515	54.0 ± 1.5^a^	29.0 ± 1.3^ab^	31.0 ± 1.2^a^	29.0 ± 1.5^a^	46.0 ± 2.2^a^
Basmati Super	63.0 ± 2.6^a^	34.0 ± 1.6^ab^	50.0 ± 2.4^a^	30 .0 ± 0.9^a^	47.0 ± 1.3^a^
Basmati S. fine	43.0 ± 1.9^a^	31.0 ± 0.6^ab^	37.0 ± 1.3^a^	27.0 ± 1.2^a^	43.0 ± 1.8^a^
Basmati Kainat	42.0 ± 0.9^a^	29.0 ± 1.6^b^	40.0 ± 1.9^a^	29.0 ± 0.8^a^	36.0 ± 1.5^a^
DPPH inhibition of rice extracts in 80% methanol					
Basmati 86	28.0 ± 0.7^a^	26.0 ± 1.1^a^	27.0 ± 0.8^a^	27.0 ± 1.1^a^	35.0 ± 1.4^a^
Basmati 515	41.0 ± 0.2^a^	30.0 ± 1.4^ab^	35.0 ± 0.8^a^	24.0 ± 0.7^a^	32.0 ± 0.7^a^
Basmati Super	48.0 ± 1.6^a^	31.0 ± 1.0^ab^	42.0 ± 1.5^a^	25.0 ± 1.3^a^	40.0 ± 1.6^a^
Basmati S. fine	38.0 ± 0.3^a^	28.0 ± 0.9^ab^	37.0 ± 1.2^a^	21.0 ± 0.3^a^	32.0 ± 0.1^a^
Basmati Kainat	28.0 ± 0.5^a^	25.0 ± 0.7^b^	27.0 ± 0.6^a^	19.0 ± 0.7^a^	22.0 ± 0.8^a^
DPPH inhibition of rice extracts in 100% acetone					
Basmati 86	41.0 ± 1.6^a^	31.0 ± 1.4^a^	39.0 ± 1.7^a^	26.0 ± 1.2^a^	29.0 ± 1.3^a^
Basmati 515	43.0 ± 1.4^a^	28.0 ± 0.5^ab^	35.0 ± 1.2^a^	24.0 ± 0.6^a^	22.0 ± 0.8^a^
Basmati Super	42.0 ± 0.6^a^	32.0 ± 0.7^ab^	36.0 ± 0.7^a^	30.0 ± 1.4^a^	30.0 ± 1.2^a^
Basmati S. fine	42.0 ± 1.5^a^	31.0 ± 1.2^ab^	32.0 ± 1.1^a^	29.0 ± 0.5^a^	31.0 ± 1.2^a^
Basmati Kainat	48.0 ± 2.2^a^	29.0 ± 1.1^b^	34.0 ± 0.9^a^	22.0 ± 0.4^a^	36.0 ± 1.4^a^
DPPH inhibition of rice extracts in 80% acetone					
Basmati 86	31.0 ± 1.1^a^	27.0 ± 0.2^a^	28.0 ± 0.8^a^	22.0 ± 1.0^a^	24.0 ± 0.5^a^
Basmati 515	27.0 ± 1.3^a^	26.0 ± 1.2^ab^	27.0 ± 1.3^a^	20.0 ± 0.3^a^	23.0 ± 0.8^a^
Basmati Super	36.0 ± 1.7^a^	31.0 ± 1.4^ab^	32.0 ± 1.5^a^	25.0 ± 0.7^a^	26.0 ± 1.1^a^
Basmati S. fine	26.0 ± 0.8^a^	22.0 ± 0.4^ab^	30.0 ± 0.8^a^	20.0 ± 1.1^a^	30.0 ± 0.9^a^
Basmati Kainat	24.0 ± 0.6^a^	22.0 ± 0.8^b^	28.0 ± 0.6^a^	21.0 ± 0.8^a^	27.0 ± 1.3^a^

*Note*: Values are mean ± SD for three samples of each variety, analyzed individually in triplicate (*n* = 3 × 3).

Means with different superscript letters with in the same row indicate significant differences (*p* < .05).

**TABLE 5:** Effect of storage on representative brown rice fatty acids composition (g/100 g)

Basmati 86				After 1st storage		After2nd storage	
S#	Acid name	Common name	Fresh	(25°C)	(5°C)	(25°C)	(5°C)
1	Tetradecanoic acid	Myristic acid	0.28 ± 0.01	BDL	BDL	BDL	BDL
2	9‐Hexadecenoic acid	Palmitic acid	17.49 ± 0.54	8.07 ± 0.25	12.87 ± 0.55	9.82 ± 0.34	10.34 ± 0.27
3	9‐Hexadecenoic acid	Palmitoleic acid	0.34 ± 0.02	BDL	BDL	17.80 ± 0.59	13.27 ± 0.52
4	Octadecanoic acid	Stearic acid	1.45 ± 0.17	BDL	BDL	3.69 ± 0.11	1.36 ± 0.04
5	9‐Octadecenoic acid	Oleic acid	38.01 ± 1.94	9.89 ± 0.37	29.53 ± 1.31	27.13 ± 0.12	27.45 ± 0.78
6	9,12‐Octadecadienoic acid	Linoleic acid	33.45 ± 1.41	BDL	26.71 ± 1.23	8.06 ± 0.31	21.91 ± 0.86
7	9,12,15‐Octadecenoic acid	Linolenic acid	2.05 ± 0.35	4.35 ± 0.13	5.01 ± 0.16	BDL	BDL
8	Pentadecanoic acid	Pentadecyclic acid	BDL	4.34 ± 0.14	2.07 ± 0.03	BDL	BDL
9	7,10‐Hexadecadienoic acid	NF	BDL	5.34 ± 0.22	0.54 ± 0.002	BDL	BDL
10	Oxiraneoctanoic acid	NF	BDL	38.05 ± 1.48	BDL	BDL	BDL
11	9‐Octadecenoic acid	Elaidic acid	BDL	BDL	BDL	6.63 ± 0.18	3.92 ± 0.09
12	Eicosanoic acid	Arachidic acid	BDL	BDL	BDL	5.58 ± 0.16	BDL
13	13‐docosanoic acid	Erucic acid	BDL	BDL	BDL	5.51 ± 0.08	2.34 ± 0.12
14	Docosanoic acid	Behenic acid	BDL	BDL	BDL	4.29 ± 0.13	BDL
Basmati Super							
1	Tetradecanoic acid	Myristic acid	1.31 ± 0.03	BDL	BDL	BDL	BDL
2	9‐Hexadecenoic acid	Palmitic acid	17.3 ± 0.72	10.72 ± 0.36	17.25 ± 0.78	10.93 ± 0.25	8.26 ± 0.26
3	9‐Hexadecenoic acid	Palmitoleic acid	0.40 ± 0.01	7.62 ± 0.29	3.51 ± 1.22	BDL	BDL
4	Octadecanoic acid	Stearic acid	3.10 ± 0.13	3.93 ± 0.16	2.07 ± 0.18	6.05 ± 0.23	3.57 ± 0.12
5	9‐Octadecenoic acid	Oleic acid	46.21 ± 2.10	21.88 ± 1.01	30.6 ± 1.43	10.93 ± 0.25	0.21 ± 0.01
6	9,12‐Octadecadienoic acid	Linoleic acid	28.64 ± 1.17	10.30 ± 0.47	20.73 ± 1.10	BDL	16.73 ± 0.64
7	9,12,15‐Octadecenoic acid	Linolenic acid	0.55 ± 0.02	BDL	BDL	BDL	BDL
8	Cyclopentanetridecanoic acid	Dihydrochaulmoogric acid	BDL	4.34 ± 0.18	2.05 ± 0.08	BDL	BDL
9	Cyclopropaneoctanoic acid	NF	BDL	8.60 ± 0.24	BDL	24.29 ± 1.07	0.21 ± 0.01
10	8,11‐Octadecadienoic acid	NF	BDL	BDL	BDL	13.39 ± 0.67	BDL
11	8‐Octadecenoic acid	NF	BDL	BDL	BDL	22.94 ± 1.12	7.12 ± 0.19
12	1,3‐Decenoic acid	Erucic acid	BDL	BDL	BDL	8.77 ± 0.31	0.01 ± 0.00
13	Docosanoic acid	Behenic acid	BDL	BDL	BDL	4.97 ± 0.21	BDL

Abbreviations: BDL, Below Detection Limit; NF, Not Found.

## 4 CONCLUSION

Awareness of the nutritional and health benefits of brown rice is important to increase the consumption in the daily human diet. After this research study, it is certain that storage of brown rice on different conditions results in significant variations in nutrients as well as antioxidant capacity. Variation in the physiochemical attributes moisture, fibers, and crude lipids for the samples stored at 25°C is significant whereas less at 5°C. Protein and ash contents remained almost same with slight nonsignificant changes. Variations in minerals content found in good agreement with that of ash contents. No significant variation observed in sugars contents at lower temperature whereas a remarkable difference found in sugar contents in the samples stored at 25°C for 3 and 6 months of storage. Antioxidant activity of rice extracts decreases up to 50% on increasing of storage period and temperature. Up to 70% decrease in the antioxidants activity observed after second storage at 25°C. The risk of nutritional change in the Basmati varieties of brown rice is reduced and its quality improves after storage at low temperatures. Therefore, it can be concluded that lower temperature is suitable to minimize the loss in the nutritional value of brown rice. Quality changes fast enough with increasing temperature. Although numerous studies have been published concerning the effect of storage conditions on rice components and qualities, information related to the impact of drying, various storage methods, and milling technologies on chemical profiling and human health benefit‐related traits (such as vitamins and antioxidant activities) is still limited and should be investigated further in future studies. Overall, Basmati kainat and Basmati super fine showed more resistance in nutritional change on storing the samples for different temperatures and duration.

## FUNDING INFORMATION

Higher Education Commission of Pakistan Project No. 8996/Punjab/HEC/R&D/NRPU/2017.

## CONFLICT OF INTEREST

The authors declare no conflict of interest.

## Supporting information


Table S1

## Data Availability

No data is available for Data Availability Statement.
