# Supplementary material for: Effect of storage on the nutritional and antioxidant properties of brown Basmati rice
Source: Food Sci Nutr. 2022 Jul 18;11(5):2086–98. doi: 10.1002/fsn3.2962 (PMC10171549; doi:10.1002/fsn3.2962)
Supplement: Supplementary file 1 — Table S1 [file FSN3-11-2086-s001.docx]

**Table S1:** Repeated measures ANOVA to identify significant variations in sugar contents of rice varieties with different storage conditions

| **Analysis of Variance for Fresh versus Sugar Varieties** | | | | | |
| --- | --- | --- | --- | --- | --- |
| **Source** | **DF** | **Adj SS** | **Adj MS** | **F** | **P** |
| Sugar | 2 | 10726 | 5362.9 | 21.77 | 0.001 |
| Varieties | 4 | 1229 | 307.2 | 1.25 | 0.365 |
| Error | 8 | 1971 | 246.4 |  |  |
| Total | 14 | 13926 |  | | |
| **Analysis of Variance for S1-25 versus Sugar Varieties** | | | | | |
| **Source** | **DF** | **Adj SS** | **Adj MS** | **F** | **P** |
| Sugar  Varieties  Error  Total | 2 | 1873.8 | 936.9 | 7.07 | 0.017 |
|  | 4 | 826.3 | 206.6 | 1.56 | 0.275 |
|  | 8 | 1060.4 | 132.5 |  |  |
|  | 14 | 3760.5 |  | | |
| **Analysis of Variance for S1-5 versus Sugar Varieties** | | | | | |
| **Source** | **DF** | **Adj SS** | **Adj MS** | **F** | **P** |
| Sugar  Varieties  Error  Total | 2 | 1895 | 947.6 | 2.10 | 0.185 |
|  | 4 | 1589 | 397.4 | 0.88 | 0.517 |
|  | 8 | 3610 | 451.3 | | |
|  | 14 | 7095 |  | | |
| **Analysis of Variance for S2-25 versus Sugar Varieties** | | | | | |
| **Source** | **DF** | **Adj SS** | **Adj MS** | **F** | **P** |
| Sugar  Varieties  Error  Total | 2 | 186.1 | 93.06 | 1.16 | 0.362 |
|  | 4 | 652.3 | 163.06 | 2.03 | 0.183 |
|  | 8 | 643.5 | 80.44 | | |
|  | 14 | 1481.9 |  | | |
| **Analysis of Variance for S2-5 versus Sugar Varieties** | | | | | |
| **Source** | **DF** | **Adj SS** | **Adj MS** | **F** | **P** |
| Sugar  Varieties  Error  Total | 2 | 3221 | 1610.4 | 3.36 | 0.087 |
|  | 4 | 1650 | 412.5 | 0.86 | 0.527 |
|  | 8 | 3836 | 479.5 | | |
|  | 14 | 8707 |  | | |
